# Development and Usability of an Inexpensive and Reusable Phantom for Ultrasound-Guided Needle Cannulation

**DOI:** 10.7759/cureus.52583

**Published:** 2024-01-19

**Authors:** Jacob Linnet, Magnús P Obinah, Mikkel H Madsen, Magnus M Møller, Lene Russell, Kim Ekelund, Morten B Svendsen, Ebbe Thinggaard

**Affiliations:** 1 Medical Education and Simulation, Copenhagen Academy for Medical Education and Simulation, Copenhagen, DNK; 2 Health and Medical Sciences, University of Copenhagen, Copenhagen, DNK; 3 Plastic Surgery, Copenhagen University Hospital, Herlev and Gentofte Hospital, Copenhagen, DNK; 4 Anesthesiology and Intensive Care, Copenhagen University Hospital, Amager and Hvidovre Hospital, Copenhagen, DNK; 5 Anesthesiology and Intensive Care, Copenhagen University Hospital, Herlev and Gentofte Hospital, Copenhagen, DNK; 6 Anesthesiology and Intensive Care, Copenhagen University Hospital, Rigshospitalet Hospital, Copenhagen, DNK; 7 Computer Science, University of Copenhagen, Copenhagen, DNK; 8 Clinical Medicine, University of Copenhagen, Copenhagen, DNK

**Keywords:** emergency medicine, anesthesiology, simulation trainer, ultrasound phantom, peripheral vein catheter, ultrasound guided

## Abstract

Introduction

Ultrasound-guided peripheral venous catheter placement (UG-PVCP) is a key skill for establishing intravenous access, especially in patients with anatomical challenges. Ultrasound is highly operator-dependent, and it is essential to ensure a sufficient level of competence when educating healthcare professionals. Competence can be acquired through simulation-based training (SBT) using phantoms or simulators. We developed a phantom for SBT, and in this study, we explore the phantom's usability and technical fidelity.

Methods

Novices with no experience in UG-PVCP and experts who routinely performed the procedure were asked to perform three ultrasound-guided catheter placement attempts on the phantom. Afterward, they were asked to complete a usability questionnaire consisting of 14 questions exploring the usability and fidelity of the phantom.

Results

Fifty-seven participants were included in the study: 29 novices and 28 experts. When assessing positive questions about the frequency of use, ease of use, integration of functionality, quickness to learn, and confidence level, the study showed a median score of 4 to 5 out of 5 in the two groups. The median was 1 to 2 out of 5 for negative questions assessing cumbersomeness, unnecessary complexity, and model inconsistency. In an additional comment textbox, one participant mentioned that the cannulation did not feel realistic but that it was good for cannulation practice.

Conclusions

We believe the phantom is suitable for an educational curriculum since it shows a high level of usability, scoring high on positive questions while scoring low on negative questions, and having high functional fidelity.

## Introduction

Establishing vascular access with a peripheral venous catheter (PVC) is an essential clinical procedure for treating hospital-admitted patients [1]. A substantial number of patients admitted to hospitals have difficult intravenous access [2]. In these patients, traditional PVC placement can require multiple attempts, leading to patient discomfort/dissatisfaction and delayed treatment and diagnostics [3]. In contrast, ultrasound-guided peripheral venous catheter placement (UG-PVCP) is associated with a high success rate of establishing vascular access in these patients [4]. Implementation of UG-PVCP in clinical settings has also been shown to reduce the use of inserted central catheters [5,6]. Ultrasound-guided procedures are known to be highly operator-dependent; therefore, an educational curriculum for UG-PVCP is needed to ensure a sufficient operator competence level [7,8]. Educational mastery learning programs using simulation-based training (SBT) with phantoms and interactive teaching have been shown to improve technical skills in medical procedures for novices without risk to patients [9,10]. Such a program could, therefore, help ensure competence and increase the implementation of UG-PVCP in clinical settings. Phantoms for UG-PVCP training are commercially available, and methods for producing do-it-yourself (DIY) versions have been described using simple remedies [11-13]. Commercial phantoms are often expensive, and DIY phantoms typically have low durability [13]. To overcome these challenges, the Copenhagen Academy for Medical Education and Simulation (CAMES) developed a low-cost and recyclable UG-PVCP phantom that can be produced using a standard 3D printer, silicone, ballistic gel, and an oven. We developed the phantom to be used in a simulation-based curriculum for UG-PVCP. This study aims to describe and explore the usability of this phantom for SBT in UG-PVCP.

## Materials and methods

Simulation model

**Figure 1:**
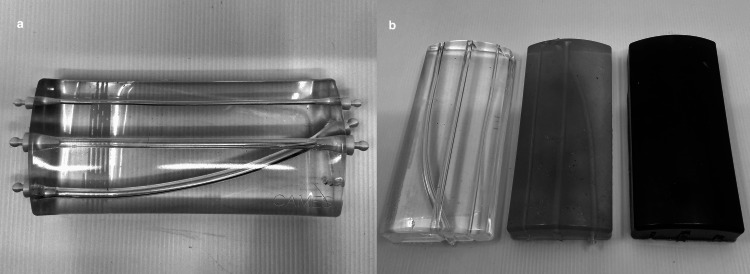
UG-PVCP phantom (a) Transparent version with visible vessels. (b) Gradual opacity achieved through color concentration variation.

CAMES developed a UG-PVCP recyclable phantom (Figure 1), focusing on learning objectives rather than high realism [14,15]. The phantom was produced using ballistic gel, a biomimicking material that allows ultrasound imaging. The gel was put into a mould, made either of aluminum using computer numerical control manufacturing (final version) or silicone using a 3D-printed scaffold (development versions). The mould was then heated in an oven at 120 degrees Celsius for three hours.

Approximately 340 g of ballistic gel (approximately 12 USD in 2022) are needed to produce one UG-PVCP phantom (refer to the Appendices section for the step-by-step production guide). The phantoms can be recycled into new phantoms after use by repeating the step-by-step guide using 340 g of ballistic gel from previously used phantoms.

The UG-PVCP phantom contained three different vessels, varying in diameter and path, for UG-PVCP training. Metal pipes were added to the mould under production to create the vessels. A similar approach has previously been described by Svendsen et al. [16] and Engberg et al. [17]. The vessels can be filled with liquid and sealed with 3D-printed plugs to mimic blood vessels when scanned.

Study design and setting

The usability survey study was conducted at a referral hospital and a university hospital in Denmark. Data was collected at the departments of anesthesiology and emergency medicine in the included hospitals. The study aimed to include two groups of participants: a novice group and an expert group of comparable size, with around 30 in each group. The inclusion criteria for novices were at least one year of experience in traditional PVC placement and no experience performing UG-PVCP. The novice group was recruited from nurses and medical students. The inclusion criterion for experts was at least one year of experience in UG-PVCP. The expert group was recruited from anesthesiologists, specialty trainees, or registrars in anesthesiology working at anesthesiology departments.

Participants were asked to fill out a participant's demography sheet (Appendices section). They were then shown a two-minute introduction video (Video 1) demonstrating the UG-PVCP procedure on the phantom using the “short-axis/out-of-plane” technique.

**Video 1:** Introduction video A step-by-step performance.

Participants equipped with a highly frequent Philips Lumify ultrasound probe (Limify L12-4 Android) connected to a tablet using the Philips Lumify Ultrasound app (c/o Philips Privacy Office (Group Legal), Amstelplein 2, 1096 BC, Amsterdam, the Netherlands) were asked to perform the procedure three times using the same technique, once for each vessel in the phantom, in the following order, with the specified remedies: UG-PVCP 1: straight, superficial vessel (6 mm), with a 1.1 mm x 32 mm 20 G PVC (pink); UG-PVCP 2: curving, profound vessel (6 mm), with a 1.1 mm x45 mm 20G PVC (pink); and UG-PVCP 3: straight, superficial vessel (3 mm), with a 0.9 mm x 32 mm 22 G PVC (blue).

Participants did not receive further guidance (neither verbally nor visually) before, during, or after the procedure. After completing the task, participants were asked to fill out a 5-point Likert scale usability questionnaire sheet (Appendices section), consisting of ten standardized questions exploring usability using the system usability scale [18] and four supplementing questions exploring technical fidelity for the UG-PVCP phantom.

Ethics and statistical analysis

No ethical approval was required according to Danish law, as the study was performed without patient involvement, but written informed consent was obtained from all participants, and participants were able to withdraw consent at any time. Statistical analysis was done using IBM SPSS Statistics for Windows, version 28 (IBM Corp., Armonk, N.Y., USA).

## Results

Fifty-seven participants were included: 29 (50.9%) novices and 28 (49.1%) experts. The median years of experience with traditional PVC placement in the two groups were nine years (range 1-35) for the novices and 14 years (range 1-40) for the experts. The median years of experience with UG-PVCP were 0 years (range 0-0) for the novices and six years (range 1-20) for the experts (Table 1).

**Table 1:** Participant demography (n=57) Values are presented as numbers and percentages. * values are presented as median and range.

	Total	Novices (n=29)	Experts (n=28)
Gender (%)			
Male	24 (42.1)	6 (20.7)	18 (64.3)
Female	33 (57.9)	23 (79.3)	10 (35.7)
Profession (%)			
Nurse	24 (42.1)	24 (82.8)	
Doctor	29 (50.9)	1 (3.4)	28 (100)
Medical student	4 (7)	4 (13.8)	
Department (%)			
Copenhagen Referral Hospital - 4,013 anesthesia	9 (15.8)	9 (31)	
Region Sjaelland University Hospital Roskilde - anesthesia	12 (21)		12 (42.9)
Region Sjaelland University Hospital Koege - emergency department	20 (35.1)	20 (69)	
Region Sjaelland University Hospital Koege - anesthesia	16 (28.1)		16 (57.1)
Experience			
Experience in PVC placement (years)		9 (range 1-35)*	14 (range 1-40)*
Experience in ultrasound-guided PVC placement (years)			6 (range 1-20)

Based on the questionnaire data assessing the usability and technical fidelity of the UG-PVCP phantom, we found a median score of 4 to 5 out of 5 in both groups when assessing the frequency of use, ease of use, integration of functionality, quickness to learn, and confidence level when handling the phantom. The median score in both groups was 1 to 2 out of 5 when assessing cumbersomeness, unnecessary complexity, and model inconsistency (Table 2).

**Table 2:** Descriptive statistics of the questionnaire

	Total		Novice		Experts	
Standardized questions	Median	Range	Median	Range	Median	Range
Q1: I think that I would like to use this model frequently	4	1-5	4	1-5	4	1-5
Q2: I found the model unnecessarily complex	1	1-4	1	1-4	1	1-4
Q3: I thought the model was easy to use	5	2-5	4	2-5	5	2-5
Q4: I think that I would need the support of a technical person to be able to use this model	2	1-5	3	1-5	1	1-5
Q5: I found the various functions in this model were well-integrated	4	2-5	5	3-5	4	2-5
Q6: I thought there was too much inconsistency in this model	1	1-4	1	1-4	1	1-3
Q7: I would imagine that most people would learn to use this model very quickly	5	1-5	5	1-5	5	3-5
Q8: I found the model very cumbersome to use	1	1-5	1	1-5	1	1-3
Q9: I felt very confident using the model	5	3-5	5	3-5	5	3-5
Q10: I needed to learn a lot of things before I could get going with this model	1	1-5	1	1-4	1	1-5
Q11: I found the PVC cannulation feeling realistic on the phantom compared to clinical practice	3	1-5	3	1-5	3	1-5
Q12: I found phantoms echogenicity of the phantom to be realistic	4	2-5	4	2-5	4	2-5
Q13: I think that the three vessels clearly demonstrated different difficulties when performing UG-PVCP placements	4	1-5	5	1-5	4	2-5
Q14: I think that training UG-PVCP placement on this phantom can be transferred to real-live UG-PVCP placement in clinical practice	5	3-5	5	3-5	5	3-5

Question 4 assessed if the participant needed assistance when using the UG-PVCP phantom. With a median score of 3 (range 1-5) among the novices and a median score of 1 (range 1-5) among the experts, there was a trend toward a more frequent need for assistance in the group of novices.

Novices and experts assessed the cannulation as feeling similarly realistic, with a median score of 3 (range 1-5). Assessing whether participants thought that skills learned on the phantom could be transferred to a clinical setting or not (Q14), the novices gave a median score of 5 (range 3-5), and the experts gave a median score of 4 (range 3-5).

Of the 57 included participants, six experts and no novices added comments in a textbox on the usability questionnaire sheet. Three of the comments addressed a lack of realism in the texture of the phantom, but one supplemented the comment by stating that the phantom was good for practicing transducer/cannulation coordination (Table 3).

**Table 3:** Comments from participants UG-PVCP: ultrasound-guided peripheral venous catheter placement

Category	Comment
Liquid in phantom vessels	Use red liquid (blood imitation) instead of clear water
Cannulations feeling in gel	The phantom is unrealistic due to higher friction than human tissue
Plastic catheter insertion	The catheter is at risk of bending when insertion after the needle is removed because of friction
Angular change when securing the catheter in the vessel	It is hard to change the angle after entering a profound vessel
Form of the phantom	The is realistic because it is rounded and mimics that of an arm
Needle/transducer coordination	Well-designed phantom to achieve skills in eye-hand coordination and needle tip visualization in UG-PVCP

## Discussion

This study investigated the usability and fidelity of a new phantom for UG-PVCP training. The study included 57 participants in total. Participants were divided into two groups: novices (n=29, 50.9%) and clinical experts (n=28, 49.1%) with extensive knowledge of the clinical procedure.

The study showed a high median score of 4 to 5 on a 5-point Likert scale when assessing the frequency of use, ease of use, integration of functionality, quickness to learn, and confidence level, and a low median score of 1 to 2 for questions assessing cumbersomeness, unnecessary complexity, and model inconsistency. This indicates that the phantom is a simple and straightforward model to use. Two questions, however, had deviating medians in the novice group. First, there was a median score of 3 (range 1-5) on the question concerning the need for assistance during the procedure, which contrasted with the same group median of 5 regarding ease of use (Q3) and one regarding the need for education before using the phantom (Q10). This finding could be because the novices have little knowledge of and no experience in UG-PVCP. We observed that a two-minute introduction video was enough to get novices going with the procedure, and most SBT programs in UG-PVCP have teachers on-site helping participants [19]. Second, the questions assessing fidelity showed a high median of 5 (range 3-5) regarding "transfer of learning" (Q14), but there was disagreement on whether the needle cannulation feeling was realistic or not with a median of 3 (range 1-5). This was also mentioned in the comment’s textbox by experts (Table 3).

Defining fidelity is challenging and ambiguous; therefore, it can be helpful to distinguish between structural realism (how simulators/phantoms appear and feel) and functional realism (what the simulator does/how the phantom works) [19]. It is important to note when designing phantoms that structural realism very often does not translate directly to educational effectiveness and focusing on functional realism instead can help reduce technical requirements and associated costs [14,15,20]. A simulator or phantom with low structural and high functional realism may even lead to more effective educational programs [19]. The UG-PVCP phantom shows some limitations on structural realism regarding cannulation feeling, but there was a high median score of 5 (range 3-5) in response to "transfer of learning," indicating a high level of functional realism and functional task alignment [19].

Several phantoms used for UG-PVCP education have previously been described in the literature [11-13]. To our knowledge, no other studies have explored the usability of the phantoms described therein, though the advantages/disadvantages of similar phantoms have been described in the literature [13]. Blue phantom (CAE Healthcare, Redmond, WA, USA), a commercial phantom, has been described as a product with homogenous echogenicity and high durability, but a tendency to accumulate needle tracks after multiple cannulation attempts and a non-tissue-like haptic [13]. Organic DIY models (i.e., models of chicken breast, tofu, and gelatin) were all described as having low durability, a long preparation time, and a risk of infection to the trainee due to their organic components [11-13]. The advantages of these DIY models are mainly their low cost, and the chicken breast was described as having realistic muscular echogenicity and needle cannulation tissue feeling [11,13]. Compared to these, the UG-PVCP phantom from CAMES is an inexpensive, recyclable DIY phantom, making it a cheap long-term option. By not being an organic product, degradation and the risk of infection are not issues.

Strengths and limitations

This study had a high number of participants, representing two potential user groups of very similar sizes. Our usability questionnaire sheet is based on a standardized form that we extended with questions assessing the technical fidelity of the phantom. Some limitations of our study are that the participants were performing the UG-PVCP and, afterward, answering the usability questionnaire sheet while on clinical duty and not in an educational setting. A shortage of time and brief introductions to the phantom may have affected the responses. Novices included were only asked about UG-PVCP experience but were allowed experience in both regular PVC and ultrasound, which may contribute to an overlap between the two included groups. The questionnaire was in English, adding to the risk of misinterpretation. Also, central tendency bias and social desirability bias are important to keep in mind when using a Likert scale questionnaire sheet.

Perspectives

We believe that our UG-PVCP phantom provides an innovative and low-cost solution for training UG-PVCP placement that can be used in future evidence-based UG-PVCP educational curricula. This will allow healthcare professionals to perform repeated training or tests without the need for live patients, reducing the risk of complications in clinical practice. From an environmental perspective, the UG-PVCP phantom is a more sustainable approach than other phantoms and can be implemented in hospitals or simulation centers that promote sustainable healthcare practices.

## Conclusions

This study using the system usability scale shows high mean scores on positive questions and low mean scores on negative questions when assessed by experts and novices. Furthermore, high mean scores were given on additional questions assessing fidelity. These results indicate high usability and fidelity for this new low-cost recyclable phantom developed for SBT of UG-PVCP. Being a simple and straightforward phantom with high functional realism, we believe it is suitable for use in future SBT of UG-PVCP.

## Appendices

**Table 4:** Step-by-step production guide

Steps	Description
1	Start by extracting the printables using the following link: https://github.com/CAMES-Engineering/UGPVC-phantom
2	Print the STL file on an FDM printer
3	Fill the entire mould with silicone
4	Remove the silicone mould from the 3D-printed mould
5	Heat an oven to 120 degrees
6	Weigh out 340 g of ballistic gel (humimic gelatin #0)
7	Fill up the silicone mould with the ballistic gel
8	Insert the silicone mould into the oven after preheating
9	After the ballistic gel has heated up and is fluent, add the aluminum tubes and the desired color
10	Wait until all air bubbles have disappeared from the silicone mould
11	Turn off the oven and wait for the ballistic gel to cool and harden
12	Carefully remove the PVC phantom from the silicone mould (use an Allen key to ensure the phantom is undamaged in the process)
13	Remove the metal tubes
14	3D-print the file Plug.stl
15	The PVC phantom is now ready for use

**Table 5:** Questionnaire: participants' demography

General information
Name
Participant number
Hospital
Department
Profession/specialty
Educational level (foundation year one, foundation year two, specialty training, specialist)
Post-graduate year (PGY)
Hand dominance (right, left, or both)
Experience in PVC placement (in years)
Experience in UG-PVCC placement (in years)
Sex

**Table 6:** Questionnaire: CAMES UG-PVCP phantom usability score CAMES: Copenhagen Academy for Medical Education and Simulation, UG-PVCP: ultrasound-guided peripheral venous catheter placement

Based on the experience of performing three UG-PVCC placements one in each of the three vessels of the CAMES UG-PVCP phantom, check the boxes based on the following statements. Don’t think too much about each statement but let each check reflect your immediate response. Ensure to respond to all statements. If you don’t know how to respond, make a check-in box “3”. Standardized question.	Strongly disagree	Disagree	Neutral	Agree	Strongly agree
	1	2	3	4	5
Q1: I think that I would like to use this model frequently	☐	☐	☐	☐	☐
Q2: I found the model unnecessarily complex	☐	☐	☐	☐	☐
Q3: I thought the model was easy to use	☐	☐	☐	☐	☐
Q4: I think that I would need the support of a technical person to be able to use this model	☐	☐	☐	☐	☐
Q5: I found the various functions in this model were well-integrated	☐	☐	☐	☐	☐
Q6: I thought there was too much inconsistency in this model	☐	☐	☐	☐	☐
Q7: I would imagine that most people would learn to use this model very quickly	☐	☐	☐	☐	☐
Q8: I found the model very cumbersome to use	☐	☐	☐	☐	☐
Q9: I felt very confident using the model	☐	☐	☐	☐	☐
Q10: I needed to learn a lot of things before I could get going with this model	☐	☐	☐	☐	☐
Q11: I found the PVC cannulation feeling realistic on the phantom compared to clinical practice	☐	☐	☐	☐	☐
Q12: I found phantoms echogenicity of the phantom to be realistic	☐	☐	☐	☐	☐
Q13: I think that the three vessels clearly demonstrated different difficulties when performing UG-PVCC placements	☐	☐	☐	☐	☐
Q14: I think that training UG-PVCC placement on this phantom can be transferred to real-live UG-PVCC placement in clinical practice	☐	☐	☐	☐	☐

## References

[1] Troianos CA, Hartman GS, Glas KE, Skubas NJ, Eberhardt RT, Walker JD, Reeves ST (2012). Special articles: guidelines for performing ultrasound guided vascular cannulation: recommendations of the American Society of Echocardiography and the Society Of Cardiovascular Anesthesiologists. Anesth Analg.

[2] Fields JM, Piela NE, Au AK, Ku BS (2014). Risk factors associated with difficult venous access in adult ED patients. Am J Emerg Med.

[3] Witting MD (2012). IV access difficulty: incidence and delays in an urban emergency department. J Emerg Med.

[4] Tran QK, Fairchild M, Yardi I, Mirda D, Markin K, Pourmand A (2021). Efficacy of ultrasound-guided peripheral intravenous cannulation versus standard of care: a systematic review and meta-analysis. Ultrasound Med Biol.

[5] Galen B, Baron S, Young S, Hall A, Berger-Spivack L, Southern W (2020). Reducing peripherally inserted central catheters and midline catheters by training nurses in ultrasound-guided peripheral intravenous catheter placement. BMJ Qual Saf.

[6] Au AK, Rotte MJ, Grzybowski RJ, Ku BS, Fields JM (2012). Decrease in central venous catheter placement due to use of ultrasound guidance for peripheral intravenous catheters. Am J Emerg Med.

[7] Moore CL, Copel JA (2011). Point-of-care ultrasonography. N Engl J Med.

[8] Hertzberg BS, Kliewer MA, Bowie JD, Carroll BA, DeLong DH, Gray L, Nelson RC (2000). Physician training requirements in sonography: how many cases are needed for competence?. AJR Am J Roentgenol.

[9] Evans LV, Dodge KL, Shah TD (2010). Simulation training in central venous catheter insertion: improved performance in clinical practice. Acad Med.

[10] Russell L, Østergaard ML, Nielsen MB, Konge L, Nielsen KR (2018). Standardised assessment of competence in focused assessment with sonography for trauma. Acta Anaesthesiol Scand.

[11] Fürst RV, Polimanti AC, Galego SJ, Bicudo MC, Montagna E, Corrêa JA (2017). Ultrasound-guided vascular access simulator for medical training: proposal of a simple, economic and effective model. World J Surg.

[12] Pollard BA (2008). New model for learning ultrasound-guided needle to target localization. Reg Anesth Pain Med.

[13] Farjad Sultan S, Shorten G, Iohom G (2013). Simulators for training in ultrasound guided procedures. Med Ultrason.

[14] Pai DR, Minh CP, Svendsen MB (2018). Process of medical simulator development: an approach based on personal experience. Med Teach.

[15] Svendsen MB, Achiam MP (2022). Defining medical simulators for simulation-based education in EUS: theoretical approach and a narrative review. Endosc Ultrasound.

[16] Svendsen MB, Ghulam QM, Zielinski AH, Lachenmeier C, Eiberg JP (2021). Validation of an assessment tool for estimation of abdominal aortic aneurysm compression in diagnostic ultrasound. Ultrasonics.

[17] Engberg M, Lönn L, Konge L (2021). Reliable and valid assessment of procedural skills in resuscitative endovascular balloon occlusion of the aorta. J Trauma Acute Care Surg.

[18] (2022). System Usability Scale (SUS). https://www.usability.gov/how-to-and-tools/methods/system-usability-scale.html.

[19] Hamstra SJ, Brydges R, Hatala R, Zendejas B, Cook DA (2014). Reconsidering fidelity in simulation-based training. Acad Med.

[20] Kneebone R (2005). Evaluating clinical simulations for learning procedural skills: a theory-based approach. Acad Med.

